# Recent advances in therapies utilizing superabsorbent hydrogel technology for weight management: A review

**DOI:** 10.1002/osp4.574

**Published:** 2021-12-07

**Authors:** Louis J. Aronne, John E. Anderson, Alessandro Sannino, Elaine Chiquette

**Affiliations:** ^1^ Division of Endocrinology, Diabetes, & Metabolism Comprehensive Weight Control Center Weill Cornell Medicine New York New York USA; ^2^ The Frist Clinic Nashville Tennessee USA; ^3^ Gelesis, Inc. Boston Massachusetts USA; ^4^ Department of Engineering for Innovation University of Salento Lecce Italy

**Keywords:** body weight, hydrogel, obesity, obesity treatment, overweight, preobesity, superabsorbent hydrogel, weight loss, weight management

## Abstract

Long‐term therapeutic benefit of treatments for weight management in patients with overweight (also termed preobesity) or obesity may be limited by variable safety, tolerability, and efficacy profiles, and patient adherence to treatment regimens. There is a medical need for nonsystemic treatments that promote weight loss in patients with overweight or early obesity. This report reviews four different approaches of utilizing superabsorbent hydrogel technology for weight management at varying stages of preclinical and clinical development. The first is a nonsystemic, oral superabsorbent hydrogel created from naturally derived building blocks used in foods (cellulose‐based), designed to mix homogenously with and change the properties of the ingested meal throughout the gastrointestinal tract (stomach and small intestine). This is the first‐in‐class to be cleared by the Food and Drug Administration (FDA) to aid in weight‐management for adults with BMI of 25–40 kg/m^2^ in conjunction with diet and exercise. In contrast, the other three approaches in development utilize superabsorbent hydrogel technologies to support an intragastric balloon‐like structure, solely occupying space in the stomach and displacing the meal: (1) a pufferfish‐inspired device; (2) Epitomee, a pH‐sensitive self‐expanding hydrogel device; and (3) a light‐degradable hydrogel used to control balloon deflation. These new approaches that utilize superabsorbent hydrogel technology offer a wide range of clinical applicability and have the potential to broaden the weight management treatment landscape. Over time, increasing the number of patients treated with superabsorbent hydrogel technologies will provide important information on long‐term efficacy and safety.

## 1 INTRODUCTION

Overweight (also termed preobesity, body mass index [BMI] 25.0–29.9 kg/m^2^) is the preceding condition that in most cases will ultimately progress to obesity.^1^, ^2^ Increased risk of morbidity and mortality are not solely restricted to higher BMI categories (Class 2 and 3 obesity, BMI 35 to <40 kg/m^2^ and BMI ≥40 kg/m^2^, respectively) but also exist in mild obesity (Class 1, BMI 30 to <35 kg/m^2^) where life expectancy is reported to be reduced by 2–4 years^1^, ^3^ While some individuals may be protected by a slightly increased body weight,^4^ others will have comorbidities including prediabetes and diabetes or will be in the process of gaining weight over their adult years on their way to obesity.^5^ Close to 39% of deaths and 36% of disability‐adjusted life years related to higher BMI occur in people with overweight, not obesity.^6^ While results from some studies have shown no association between abnormal weight gain or overweight status and greater morbidity and mortality,^7^ data from the Framingham study demonstrated that nonsmoking women who are aged 40 years with overweight lose 3.3 years of life, and nonsmoking men who are aged 40 years with overweight lose 3.1 years of life compared with age‐matched individuals who are at normal weight.^8^ Obesity‐related diseases/comorbidities are associated with loss of between 0.2 and 11.7 years of life, depending on an individual's sex, race, BMI classification, age, and heightened risk of mortality.^9^ In some people with overweight, continued progressive weight gain may lead to further health risks and eventually obesity; this holds true for older populations with obesity‐related diseases, where a loss of lean body mass or muscle mass can be outpaced by a gain in fat mass.^5^, ^10^, ^11^ In the elderly population, a BMI in the overweight range may falsely indicate lower risk.

The long‐term therapeutic benefit of current treatment options (behavioral, nutrition, and lifestyle approaches; pharmacotherapy in conjunction with comprehensive lifestyle management; procedural medical devices, [e.g., intragastric balloon such as the TransPyloric Shuttle]; noninvasive endoscopic procedures; and bariatric surgeries [e.g., Roux‐en‐Y gastric bypass]) is limited by variable efficacy, safety, and tolerability profiles and patient adherence.^12^, ^13^, ^14^, ^15^, ^16^, ^17^ Long‐term weight loss maintenance is difficult to achieve and maintain with lifestyle modification alone.^18^, ^19^ There are multiple barriers to effective long‐term weight management care,^20^ including patients' lack of access to healthcare for weight management,^21^, ^22^ clinical inertia that prevents providers from offering care,^23^, ^24^, ^25^ the costs of treatment, patients' inability to comply with a weight management plan, and the problem of long‐term efficacy of current weight‐loss treatment options.^26^, ^27^, ^28^ The worldwide surge in obesity prevalence dictates the need to increase the availability of therapeutic strategies that alter the course of the disease.^29^, ^30^, ^31^ Interestingly, the prevalence of overweight is actually declining as obesity develops in more people at a greater rate.^27^ There is a medical need for therapies that result in clinically meaningful weight loss with favorable safety profile, that will allow patients to be treated earlier while they are at a lower BMI with the hope of preventing or delaying the onset of complications related to overweight and obesity.

Interest is developing around the utilization of superabsorbent hydrogel technologies as a nonsystemic, biodegradable approach for weight management, with the idea that achievable weight loss and maintenance in individuals in the overweight range can prevent development of greater levels of obesity and attendant comorbidities.^32^, ^33^, ^34^ The following is a review of the emerging science and description of several approaches utilizing superabsorbent hydrogel technologies at varying stages of preclinical and clinical development.

## 2 THERAPIES UTILIZING SUPERABSORBENT HYDROGEL TECHNOLOGY FOR WEIGHT MANAGEMENT

A superabsorbent hydrogel is a cross‐linked polyelectrolyte polymer that is capable of absorbing and retaining large quantities of fluid/water (up to more than 1 L/g of dry material without losing its three‐dimensional [3‐D] structure under moderate compression).^35^ Superabsorbent hydrogels appear as a fine white powder‐like sand or tiny granule‐like sugar in a dry state and transform into 3‐D structures filled with water once fully hydrated. Hydrogels can be classified based on their natural or synthetic origins, type of cross‐linking (chemical or physical), physical appearance (sphere, matrix, or film), and electrical charge (nonionic, ionic, etc.).^36^ According to the required application, hydrogels can be tailored to absorb more or less water, to be more or less rigid/firm, and to be responsive to external stimuli such as the ambient temperature, pH, light, ionic strength, or electric field.^36^ Because of their tissue‐like softness and heightened biocompatibility, hydrogels are widely utilized in biomedical applications, including enhanced drug delivery systems, tissue engineering, and wound healing, in addition to weight loss therapies.^34^, ^37^, ^38^

## 3 FDA‐CLEARED ORAL SUPERABSORBENT HYDROGEL FOR WEIGHT MANAGEMENT

**FIGURE 1:**
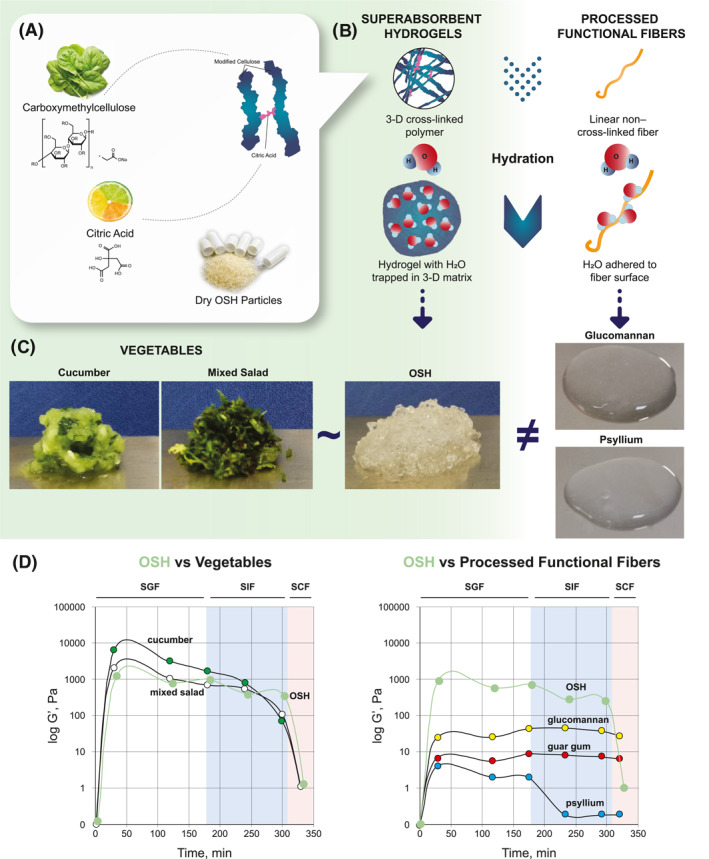
Comparison of viscoelastic properties of superabsorbent hydrogel versus processed functional fibers and vegetables rich with natural fiber. (A) OSH is made up of modified cellulose (carboxymethylcellulose) and citric acid found in plant cell walls and citrus fruits, respectively. Dry OSH particles are each approximately the size of a grain of salt. (B) Superabsorbent hydrogels are cross‐linked polymer networks that trap water in a three‐dimensional matrix. Because functional fibers have a linear, non–cross‐linked structure, water adheres to the surface of the fiber. Superabsorbent hydrogels can absorb much larger quantities of fluids than functional fibers, thus resulting in more rigid, elastic gel particles. (C) Visual comparison of vegetables, OSH, and processed functional fibers. OSH is more like vegetables than processed functional fibers. (D) Comparison of elastic modulus (log‐transformed G′ values in pascals) between OSH and vegetables (graph on left) and between OSH and processed functional fibers (graph on right). OSH G′ pattern is consistent with masticated mixed salad greens and cucumber as opposed to processed functional fibers (glucomannan, guar gum, and psyllium). 3‐D, 3‐dimensional; OSH, oral superabsorbent hydrogel; SCF, simulated colonic fluid; SGF, simulated gastric fluid; SIF, simulated small intestine fluid

Plenity^®^ (Gelesis, Inc.) is a novel, orally administered, nonsystemic, superabsorbent hydrogel, and the first in its class to be cleared by the Food and Drug Administration (FDA) to aid in weight management for adults with a BMI of 25–40 kg/m^2^ in conjunction with diet and exercise.^39^, ^40^, ^41^ Each capsule contains thousands of dry, oral superabsorbent hydrogel (OSH) particles, and each particle is approximately the size of a grain of salt (100–1000 μm). The OSH particles are made up of carboxymethylcellulose (a derivative of cellulose, the main substance in plant cell walls) cross‐linked with citric acid, both of which are food grade and generally recognized as safe (Figure 1A). To our knowledge, OSH is the first and only superabsorbent hydrogel therapy produced using solely nonsynthetic, naturally derived building blocks. Additionally, OSH is a hydrogel that reacts to its environment.

**FIGURE 2:**
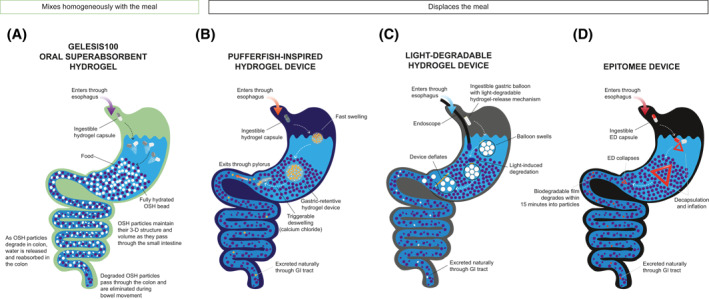
Comparison of mechanism of action of OSH, pufferfish‐inspired hydrogel, light‐degradable hydrogel, and ED. Comparison of mechanism of action of OSH (A), the pufferfish‐inspired hydrogel (B), a light‐degradable hydrogel device (C), and ED (D). Note, both endoscopic and ingestible light sources can be used to trigger degradation of the hydrogel‐release mechanism of the light‐degradable hydrogel device. Volume is dependent on the fluid absorption capacity of the hydrogel. Elasticity is the ability of a material to maintain its normal shape after being compressed. Time to hydrate is the amount of time required for hydration and maximizing elasticity. Degradation is the rate of dissociation of the hydrogel and release of fluid in the colon. ED, Epitomee device; GI, gastrointestinal; OSH, oral superabsorbent hydrogel

OSH is intended to be taken as three capsules (0.75 g each) with 500 ml of water, 20–30 min before lunch and dinner.^39^ In the stomach, the capsules disintegrate and release thousands of OSH particles (Figure 2A). Each particle responds to changes in gastrointestinal pH and ionic milieu and quickly expands by absorbing water. Individual, fully hydrated beads (each approximately 2 mm in diameter) do not cluster or form a large mass but rather mix homogenously with food to change the texture, rheologic behavior, and sensory characteristics of the meal.^42^ A meal mixed with individually hydrated, solid, 3‐D beads increases in volume (approximately 250 ml larger volume), viscosity/thickness, and firmness, without increasing calories. The hydrated beads are resistant to digestion and maintain their 3‐D structure and mechanical properties (increasing volume, viscosity, and firmness of the content) through the small intestine.^39^ The stomach and gastrointestinal tract are highly regulated organs with complex neural and hormonal control mechanisms.^43^ The external muscle layers of the gastrointestinal tract are sensitive to stretch (sensing an increase in volume) and tension (sensing an increase in firmness) and this mechanical effect may result in activation of the vagus nerve which in term could trigger signals of satiety and satiation.^44^, ^45^ The OSH particle remnants are not digested, metabolized, or absorbed during intestinal transit, have no nutritional value, and are naturally degraded and eliminated from the body in the feces.^39^ OSH is considered an encapsulated medical device and is regulated by the FDA as such because its primary function is achieved through mechanical modes of action and its effects are nonsystemic in nature.^34^, ^46^

Once fully hydrated, OSH exhibits rheologic behavior that is markedly different from dietary fiber supplements; OSH forms a solid, while dietary fibers (e.g., glucomannan, guar gum, and psyllium) are viscous liquids (Figure 1B).^42^ While water adheres to the surface of dietary fibers, which have a linear, non–cross‐linked structure, the cross‐linked polymers that make up OSH trap water in a 3‐D sphere of more rigid, elastic solid beads. OSH (solid, form filled with water) more closely resembles natural fiber‐rich vegetables (e.g., cucumber and mixed green salad) than dietary fiber (Figure 1C). While dietary fibers may increase the viscosity/thickness of the meal, OSH, like natural fiber‐rich vegetables, exhibits combined properties of increased viscosity, and firmness/elasticity and therefore changes the mechanical properties of the meal.

The higher the degree of cross‐linking, the lower the swelling capacity (i.e., fluid absorption capacity) and the higher the elasticity (i.e., ability of a material to maintain its normal shape after being compressed) of the hydrogel. Results from a study of gastrointestinal digestion using an in vitro model demonstrated that the viscoelastic properties of OSH were comparable to those of masticated cucumber and mixed green salad (i.e., natural fiber‐rich vegetables) and were orders of magnitude higher to common processed dietary fiber supplements (e.g., glucomannan, guar gum, and psyllium) (Figure 1D).^42^ These data provide further insight into the mechanisms underlying the weight‐loss outcomes in patients treated with OSH technology (detailed below).

As reported by Greenway et al.,^34^ results from the multicenter, randomized, double‐blinded, placebo‐controlled, pivotal Gelesis Loss of Weight (GLOW) study (NCT02307279) demonstrated that OSH is effective, safe, and well tolerated for weight loss in patients with excess weight (overweight [BMI 25–29.9 kg/m^2^] or obesity [BMI ≥30 kg/m^2^]). A total of 436 patients were randomized in the GLOW study (223 to OSH and 213 to placebo); and the co‐primary efficacy endpoints were the percent change in body weight and the percent of patients who lost ≥5% body weight from baseline, both from baseline to day 171. OSH capsules were taken two times per day, 20–30 min before lunch and dinner (three capsules per dose). About 6 of 10 patients (59%) taking OSH achieved significant and clinically meaningful weight loss (5% or more), and 27% of patients lost 10% or more of their total body weight (∼30 pounds). Comparatively, 42% and 15% of patients on placebo had ≥5% and ≥10% weight loss, respectively. In a post hoc analysis, early weight loss predicted long‐term effectiveness of OSH (i.e., 3% or more weight loss at 8 weeks was predictive of weight loss of 5% or more after 6 months). No weight loss plateau was observed during the 6‐month GLOW study, and weight loss was sustained during the 24‐week follow‐up period.^34^ The overall incidence of adverse events (AEs) was no different than placebo. The most common treatment‐related AEs reported in patients taking OSH were gastrointestinal related (i.e., diarrhea, abdominal distension, infrequent bowel movements, and flatulence). OSH does not affect the absorption of vitamins and minerals after 24 weeks of administration,^34^ and the effect of OSH on metformin absorption is the same as that of a typical meal.^47^

### 3.1 Encapsulated gastric space‐filling devices utilizing superabsorbent hydrogel technologies in development

Based on the defense mechanism of pufferfish that quickly inhale large amounts of water to inflate, Liu et al.^33^ developed a noninvasive, superabsorbent hydrogel‐based device from two synthetic polymers. The inner material contains sodium polyacrylate (polyacrylic acid particles [∼450 μm in diameter]) that are superabsorbent particles used in commercial products such as diapers for their ability to rapidly soak up liquid and inflate. To prolong longevity in the stomach, a second protective anti‐fatigue porous hydrogel layer encapsulates the superabsorbent particles. The superabsorbent hydrogel particles are encapsulated, delivered orally (i.e., ingested as a standard‐sized pill ∼1 cm in length), and can swell ∼100 times in volume in one large “ball” (Figure 2B). The resulting sphere is ∼6 cm in diameter, large enough to avoid passing through the pylorus and strong enough to allow long‐term residence in the stomach, while markedly softer than an intragastric balloon to minimize mucosal damage. It is conceivable that for a weight‐loss indication, patients could take several capsules that would swell to the size of several golf balls. Because of the potential risk of migration to the intestine and possible risk for bowel obstruction, the sphere can be rapidly deflated when submerged in a solution of calcium chloride (i.e., giving a glass of milk supplemented with calcium). Results from a preclinical study performed in a validated porcine model demonstrated long‐term robustness of the hydrogel device, which can be retained in the gastric environment up to 29 days in vivo, while maintaining mechanical properties and performance.^33^ To date, no clinical trials are reported.

More recently, Raman and colleagues^48^ introduced the first biocompatible, light‐degradable hydrogel with tunable mechanical properties as a device for in vivo gastrointestinal applications (Figure 2C). Chemically synthesized from a light‐cleavable, acrylated *ortho*‐nitrobenzyl (oNB)–based moiety linker (responsive to 365‐ to 405‐nm blue light) that polymerizes 3‐D networks of light‐unresponsive poly(acrylamide) and poly(2‐acrylamido‐2‐methyl‐1‐propanesulfonic acid), this dynamic hydrogel can be functionally applied to create light‐triggerable, gastric space‐filling devices (e.g., an oral intragastric balloon). The hydrogel is cast in a capped pin shape and utilized to seal the open end of a gastric balloon, which is made of a porous polymer stuffed with a filler that rapidly inflates when wet (Figure 2C). For the balloon to deflate on demand, Raman and colleagues demonstrate that either an endoscope fitted with an LED light or a battery‐powered LED capsule capable of magnetic docking with the balloon, may be used to activate the degradation of the hydrogel‐based pin, thus driving the leakage of the filler and the volumetric reduction of the balloon. Results from preclinical porcine studies validated that oNB cross‐linked hydrogels are preliminarily safe for in vivo applications^48^

The Epitomee device (ED; Epitomee Medical) is an orally self‐administered device under investigation for weight loss in patients with overweight or obesity. The ED is a folded, encapsulated, superabsorbent multilayered film made from pharmaceutical‐grade polymers and bonding materials that unfold and self‐expand in the stomach. The superabsorbent core layer of the ED may absorb up to 100 times its dry weight, while the flexible external layer is designed to provide resistance in the gastric environment. On uptake of liquid, the ED unfolds and shapes into a 3‐D solid with large size (Figure 2D) and high rigidity/firmness (∼15–150 kPa), close to that of inflated intragastric balloons.^49^ The unfolded ED is pH‐sensitive and can reside in the stomach for a given time (<24 h), after which it is reported to collapse and travel to the small intestine, where it is, within 30 min, completely degraded to particle form. The ED works as a transient gastric space‐filling device, similar to the pufferfish‐inspired device in development discussed above. Shirin et al.^32^ recently reported results from an open‐label, single‐center, single‐arm, phase 2 study (NCT03610958) in patients (aged 25–61 years, BMI 27–40 kg/m^2^, with or without controlled hypertension and/or dyslipidemia) demonstrating that ED treatment combined with lifestyle modification led to 3.7%–4.5% total body weight loss after 3 months. In addition to reductions in BMI and waist circumference, 31% of the total population and 42% of study completers achieved 5% or more total body weight loss after 3 months of treatment with ED. In this study, patients swallowed one ED capsule with two cups of water twice per day approximately 30 min prior to lunch or dinner and were instructed to follow a hypocaloric diet. ED was highly tolerable with a favorable safety profile. The most commonly reported AEs were headache, viral infection, abdominal discomfort, bloating, nausea, constipation, and flatulence.^32^ Results from an endoscopic examination revealed mild, asymptomatic gastric/duodenal erythema without erosions or need for medical intervention in five of 26 patients (19%).^32^ A prospective, randomized, double‐blind, placebo‐controlled, multicenter, pivotal, adaptive study of the effect of ED on body weight in patients with overweight and obesity with and without prediabetes is currently recruiting participants (NCT04222322).

The rheologic properties and mechanosensory effects of OSH differs from gastric space filling devices. OSH elasticity is similar to vegetables (2 kPa); instead of forming one large sphere, the individual OSH beads mix homogeneously with the meal (Figure 2A), adding volume without adding calories while restoring the meal's mechanical properties and mimicking a meal filled with fiber‐rich vegetables. OSH mechanical effects are not restricted to the stomach. As food is digested and pH decreases, the individual OSH beads shrink and are released into the small intestine where they re‐expand in the presence of a higher pH. Intragastric balloons or hydrogels mimicking balloons exert their effect solely in the stomach by displacing the meal volume leading to exaggerated gastric distension.^42^, ^50^, ^51^ OSH administration leverages the intermittent food intake at mealtimes to stimulate mechanosensory receptors only at the time of meals, thus minimizing the risk of desensitization.

### 3.2 Other potential clinical applications for oral superabsorbent hydrogel

Looking beyond the satiety mechanism of OSH, several preclinical studies have demonstrated broad applicability of the OSH technology on metabolic health via intestinal mechanisms. In a mouse model of severe intestinal injury, Gelesis OSH restored function of the gut barrier similar to that of the naïve control group.^52^ In a high‐fat diet‐induced nonalcoholic fatty liver disease mouse model, superabsorbent hydrogel technology reduced insulin resistance and increased glucagon‐like peptide‐1 levels, improved gut barrier function, and modified gut microbiota composition.^53^ Thus, available data from the mouse model suggest that this technology may be a potential therapeutic option to restore gut homeostasis for metabolic diseases, such as diabetes and nonalcoholic fatty liver disease^53^; however, clinical data are needed to validate these findings.

## 4 DISCUSSION

Current interventions for weight management in patients with obesity are limited by variable safety, tolerability, efficacy profiles, and patient adherence. There is a medical need for nonsystemic treatments that are effective and safe at promoting weight loss and/or preventing further weight gain in patients with overweight or obesity. In the same way that clinicians treat moderately high blood pressure early on before the onset of pathological hypertension, treatment of overweight is a viable strategy to alter the course of obesity disease.

Different approaches of utilizing superabsorbent hydrogel technology offer a wide range of clinical applicability and the potential to broaden the treatment landscape for patients with overweight, obesity, or obesity‐related metabolic diseases. Of the therapies that are at different stages of preclinical and clinical development, OSH is the one cleared thus far by the FDA to aid weight‐management in adults with a BMI of 25–40 kg/m^2^ in conjunction with diet and exercise. Treatment with OSH can result in clinically meaningful weight loss, and given its favorable safety and tolerability profile, there appears to be limited increased risk associated with the use of OSH to support weight management. Therefore, OSH opens the possibility to treat patients earlier while at a lower BMI.

## CONFLICT OF INTERESTS

Louis J. Aronne has received funding/grant support/honorarium from Gelesis, Myos Corporation, Jamieson Wellness, Novo Nordisk, Pfizer, Zafgen Inc., Intellihealth, ERX, Astra Zeneca, Sanofi, Janssen, Pfizer, and UnitedHealth Group. He holds stock or stock options in Jamieson Wellness, Intellihealth, Zafgen, and Gelesis. John E. Anderson has received honorarium for consultancy/advisory boards/speakers bureaus from Eli Lilly, Boehringer Ingelheim, Sanofi, Novo Nordisk, Astra Zeneca, Janssen, Abbott Diabetes, and Mannkind. Alessandro Sannino and Elaine Chiquette are employees/consultants of Gelesis and may hold stock or stock options.
